# Pain Intensity Is Not Always Associated with Poorer Health Status: Exploring the Moderating Role of Spouse Personality

**DOI:** 10.1155/2018/7927656

**Published:** 2018-09-26

**Authors:** Carlos Suso-Ribera, Michael J. L. Sullivan, Santiago Suso-Vergara

**Affiliations:** ^1^Department of Basic and Clinical Psychology and Psychobiology, Jaume I University, Castellón de la Plana, Spain; ^2^Department of Psychology, McGill University, Montreal, Canada; ^3^Department of Traumatology, Clinic Hospital, Barcelona, Spain

## Abstract

**Background:**

Past decades have seen a surge of studies investigating the role of spouses in chronic illness. The present study explored an interpersonal model of health-related quality of life in chronic pain settings. Spouse personality was tested as a moderator of pain intensity-to-health associations in patients with chronic pain.

**Methods:**

This is a cross-sectional study. Participants were 185 noncancer chronic pain patients and their spouses. Patients were mostly females (58.4%). Mean age was approximately 56 years for patients and spouses. Patients completed a measure of pain intensity, health-related quality of life, and personality. Spouses also reported on their personality characteristics. Spouse personality was used as the moderator in the relationship between patients' pain intensity and health status. Patient personality was used as a covariate in the moderation analyses.

**Results:**

Spouse neuroticism moderated the relationship between pain intensity and physical health status, while spouse introversion moderated the pain-to-mental health association.

**Conclusions:**

Results support the idea that the relationship between a chronic stressor, namely, chronic pain, and health-related quality of life may be complex and contextually determined by spousal characteristics. Clinical implications are discussed in the context of couples.

## 1. Introduction

Chronic pain is a common and disabling disease [1–3]. Similar to other chronic illnesses [4, 5], spouses of pain patients have been argued to play an important role in the disease due to their caregiving role [6]. In chronic pain, spouse responses to patients' pain behavior have been the focus of most research [7], while the influence of spouse personality characteristics has been mostly overlooked. This is surprising because personality traits have been consistently associated with mental and physical health status across healthy and sick populations, including chronic pain [8, 9].

Personality can be defined as relatively stable ways in which people think, feel, and behave [10]. The five-factor model of personality (FFM), which describes personality in terms of five dimensions (neuroticism, extraversion, agreeableness, openness to experience, and conscientiousness), is currently the dominant framework for personality. In the FFM, neuroticism is understood as a tendency to be emotionally unstable and to experience negative emotions (i.e., anxiety, depression, and anger). Neuroticism is considered a risk factor for health as it has been associated with poorer physical functioning, increased worry about symptoms, and greater mental distress. Conversely, extraversion and conscientiousness tend to be associated with better health outcomes, including physical functioning and mental well-being. Extraverts tend to be optimistic, physically active, and socially competent, while conscientiousness is associated with high self-discipline, dutifulness, and low risk taking. Agreeableness and openness, which are the socioaffective and intellect dimensions, respectively, have the less consistent and weakest associations with health outcomes [9, 11, 12].

Consistent with the literature on neuroticism, when spouse interaction styles are characterized by negative emotionality (i.e., preoccupied and fearful or angry and critical), patients tend to report increased mental distress. By contrast, the relationship between these negative responses and pain intensity and physical disability levels is weaker and inconsistent, and the role of arguably positive spouse behaviors, such as solicitous and validating responses, is still inconclusive [13–17]. One limitation of existent research is that spouse factors are argued to have a direct effect on patient outcomes. However, it is also possible that spouse characteristics influence patients' status by moderating (i.e., reducing or aggravating) the impact that pain has on health [7]. This is consistent with the Transactional Model Of Health, which emphasizes that challenges of the painful condition occur in a social (family) context and argues that spouse factors can improve or exacerbate stressors [18].

There is, indeed, some support for this contextual (i.e., moderating) role of spouse personality in the context of couples. For instance, a study showed that spouse neuroticism influenced the associations between everyday problems and affect and physical symptoms in older couples [19]. In another investigation, the relationship between patient personality and patient psychological health differed as a function of spouse personality [20]. What these studies suggest is that the relationship between stressors (i.e., everyday problems and certain personality styles) and patient health can be influenced by spouse characteristics. The extent to which spouse personality can also be an important contextual factor in the presence of a chronic stressor, such as pain, remains unexplored.

The present study aims at testing whether the negative influence of patient pain (stressor) on patient health-related quality of life (outcome) is moderated by psychological factors in the spouse (e.g., spouse personality; contextual factor). Research has consistently shown that health-related quality of life decreases with pain [21, 22]. Based on previous research showing that neuroticism, introversion, and low conscientiousness are also associated with poor health status [9], we expect that the aforementioned spouse psychological characteristics will impose low health-related quality of life in pain patients irrespective of pain levels (i.e., the burden of pain is increased).

## 2. Methods

### 2.1. Design, Participants, and Settings

This observational study was conducted at the Pain Clinic of the Valld'Hebron Hospital in Barcelona, which is a tertiary care pain clinic. This clinic was selected because it was the home institution of the corresponding author, C. S. R., when the study was conducted. Also, past research by our team has revealed that patient characteristics at this clinic are comparable to those of other tertiary pain clinics [23], so the results obtained might be applicable to a considerable number of pain clinics.

Eligibility criteria included (i) having a diagnosis of chronic noncancer pain (>3 months of duration), (ii) having an appointment at the Pain Clinic of the Valld'Hebron Hospital between January 2014 and December 2015, and (iii) being married at the time of assessment. Consecutive sampling was used for recruitment, which started in January 2014 and finished in December 2015. One month prior to the patients' appointment at the Pain Clinic, the lead researcher, C. S. R., explored the eligibility criterion of having a diagnosis of chronic pain in the electronic medical records and ICD-9 code 338.2. Patients meeting this criterion were called to explore the eligibility criterion of marital status. If they were married and willing to participate in the study, two letters were sent to the patient's home, one for the patient and the other for the spouse. For both, each letter included a description of the goals, procedures, and possible risks of participating in the study, together with the contact information of the lead researcher, C. S. R., the informed consent form, and the questionnaires.

Couples were asked to complete the forms separately and were given an envelope that had to be sealed and returned to the physician the day of the patient's first visit to the Pain Clinic. All participants provided their written consent to participate in the study and did not receive any economic compensation for their participation.

The Ethics Committee of the Vall d'Hebron Hospital in Barcelona approved the current study.

### 2.2. Measures

#### 2.2.1. Spouse Personality

The NEO five-factor Inventory (NEO-FFI) [10] evaluates five dimensions of adult personality, namely, neuroticism, extraversion, openness to experience, agreeableness, and conscientiousness. Of these, only neuroticism, extraversion, and conscientiousness appear to be consistently associated with health outcomes in chronic pain settings [8, 9, 12]. However, because this is a novel approach to the role of personality in chronic pain, the moderating role of openness and agreeableness will also be investigated.

In the NEO-FFI participants rate their degree of agreement on a 5-point Likert scale (0 = *totally disagree*; 4 = *completely agree*). Each of the five personality dimensions is composed of 12 items, so scores for each dimension range from 0 to 48. The internal reliability of the personality dimensions in our study (0.64 < *α* < 0.84) was comparable to previous findings (0.66 < *α* < 0.81) [10, 24].

#### 2.2.2. Patient Pain Intensity

Current pain intensity was assessed with a Numerical Rating Scale, which has become a standard in the measurement of pain [25] and is widely recommended due to its compliance rate, responsiveness, and ease of use [26]. Participants in our study rated their pain from 0 = *no pain* to 10 = *worst possible pain*.

#### 2.2.3. Patient Health-Related Quality of Life

Physical and mental components of quality of life were assessed with the Short Form-36 Health Survey [27], which has become one of the most widely used instruments for the assessment of health-related quality of life [28]. Physical aspects include the ability to perform daily activities (Physical Functioning) and work-related activities (Role Physical), plus the average intensity of pain in the last four weeks (Bodily Pain). Some elements correlate to physical and mental health, such as the perception of present and future health (General Health), the evaluation of personal energy (Vitality), and the interference of health problems in their interpersonal life (Social Functioning). The remaining components, namely, the role of emotions on functioning (Role Emotional) and psychological well-being (Mental Health), mainly reflect psychological aspects of health [21]. A composite score can be obtained for physical and mental health using the aforementioned subscales. The use of these composite scores is often recommended for methodological reasons [29]. However, the use of the physical composite in the present study would be problematic because it contains a pain subscale, which would contaminate the relationship between the dependent variable (i.e., health) and the independent variable (i.e., pain intensity). Thus, physical health was measured by means of the Physical Functioning scale. The Mental Composite Score is not contaminated by the presence of pain intensity ratings, so it was used to assess overall mental health to reduce the number of statistical tests. Scales and composite scores in the Short Form-36 have a 0–100 range. High scores represent better functioning. The internal consistency in our study (0.86 < *α* < 0.95), which is calculated for the 8 subscales, was consistent with previous findings (0.78 < *α* < 0.94) [30].

#### 2.2.4. Covariates

Patient age, sex, pain duration, educational level, and personality were used as covariates due to their relationship with patient health status [8, 31]. Patient education was coded as 0 = “less than 12 years of education” and 1 = “more than 12 years of education.” Patient personality was assessed with the NEO-FFI, the same measure that was used to evaluate spouse personality.

### 2.3. Statistical Analyses

The moderating effect of spouse personality on the relationship between pain and health-related quality of life in the patient was tested using multiple linear regressions. In the regressions, patient sex, age, and personality were included as covariates in the first block. Patient pain intensity was entered next. The third and the forth blocks included spouse personality and the interaction term (patient pain intensity ∗ spouse personality), respectively. Multiple regression diagnostics included an analysis of multicollinearity (i.e., two predictors are linear combinations of one another) and a test of unusual and influential data (i.e., whether certain observations are responsible for the results).

All analyses were computed using SPSS version 22 [32].

## 3. Results

### 3.1. Sample Characteristics

From January 2014 to December 2015, 515 phone calls were made. In total, 203 patients met the eligibility criteria, so a letter was sent to these patients and their spouses. Eighteen patients did not return the protocol (either them or the spouse lost interest in the study). The final sample comprised 185 chronic pain patients and their spouses (91.1% response rate). All couples were heterosexual. Patients had an average age of 56.55 years (*SD* = 13.59) and were mostly females (58.4%). The mean age for spouses was 56.66 years (*SD* = 13.85) and 41.6% were females. Approximately 94% of participants were born in Spain. Academic degree was similar for both samples, with approximately half of the participants having achieved more than 12 years of education (47.3% of patients and 50.3% of spouses). Patients had been suffering pain for an average of 6.55 years (*SD* = 8.57). Pain was mostly located in the back (58.8%) and neck (11.1%).

Means and standard deviations of study variables are presented in Table 1. Mean pain intensity was 7.71 (*SD* = 1.56) with a range of 3 to 10. The sample would be characterized as experiencing moderate to severe pain [33]. Scores for patients' physical and mental health status were comparable (between +1 *SD* and −1 *SD*) to those of previous investigations assessing similar pain populations [34–36]. Compared with population norms, mean values on physical and mental composite scores were, respectively, 2 *SD* and 1 *SD* below the mean of the general population in Spain [37]. Pain patients in this sample were significantly more physically disabled and psychologically distressed than the general population. Mean scores for spouse and patient personality were comparable (within 1 *SD*) with previously reported scores in the general population in Spain [24, 38].

### 3.2. Moderation Analyses

Before performing the moderation analyses, all predictors were centered. Patient openness and agreeableness were not used as covariates due to their weak associations with patient health-related quality of life and to reduce multicollinearity problems.

Spouse neuroticism moderated the association between patients' pain intensity and patient Physical Functioning (Table 2), while spouse extraversion moderated the pain-to-mental health relationship (Table 3). In a moderation analysis, this should be interpreted as revealing that the contribution of the independent variable (i.e., patients' pain intensity) on the dependent variable (i.e., physical and mental health) varies among different levels of the moderator (i.e., spouse neuroticism and extraversion).For example, the positive interaction coefficient between spouse neuroticism and patient pain intensity in the prediction of physical functioning (*β* = 0.19, *p*=0.009, 95% CI = 0.08, 0.54) means that patient pain intensity is less intensely associated with physical functioning at higher levels of spouse neuroticism. On the contrary, the negative coefficient in the interaction between extraversion and pain intensity in the prediction of mental health (*β* = −0.17, *p*=0.014, 95% CI = −0.30, −0.03) reveals that patient pain intensity is less intensely associated with mental health at low levels of spouse extraversion. No moderation effect was found for spouse openness to experience, agreeableness, and conscientiousness.

**Figure 1:**
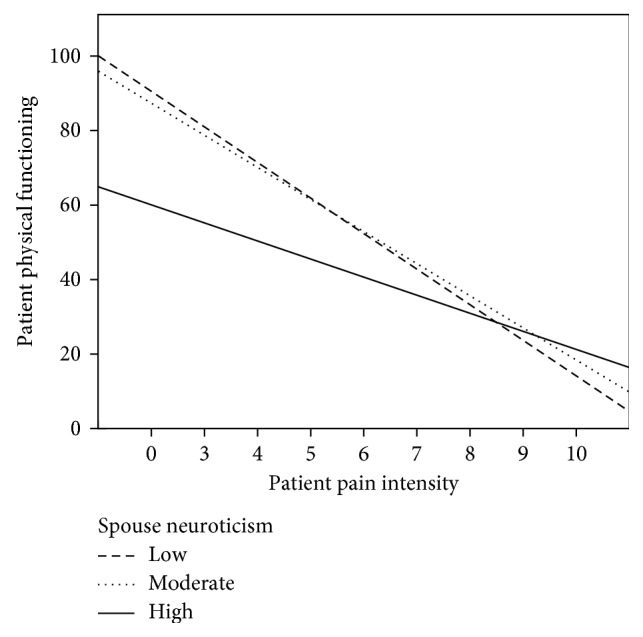
Neuroticism as a moderator of the relationship between pain intensity and Physical Functioning.

Post hoc analyses were then performed to explore if the simple slopes were significant and in the expected direction. Simple slopes are often calculated at one standard deviation from the sample mean, but its use has been argued to be arbitrary and sample specific, which might limit the generalizability of the findings (i.e., what is high in one sample might be moderate or low in another sample) [39]. Thus, we plotted the moderation using the Spanish population norms for low (percentile 30) and high (percentile 65) neuroticism and extraversion, which are 16 and 23 for neuroticism and 28 and 35 for extraversion [40]. As reflected in Figure 1, the relationship between patient pain intensity and patient physical functioning at daily activities was strongest when spouse neuroticism was low (*β* = −0.47, *t* = −4.22, *p* < 0.001; 95% CI = −14.08, −5.03) or moderate (*β* = −0.55, *t* = −4.67, *p* < 0.001; 95% CI = −12.31, −4.90), as opposed to high (*β* = −0.33, *t* = −2.82, *p*=0.006; 95% CI = −7.17, −1.23).

**Figure 2:**
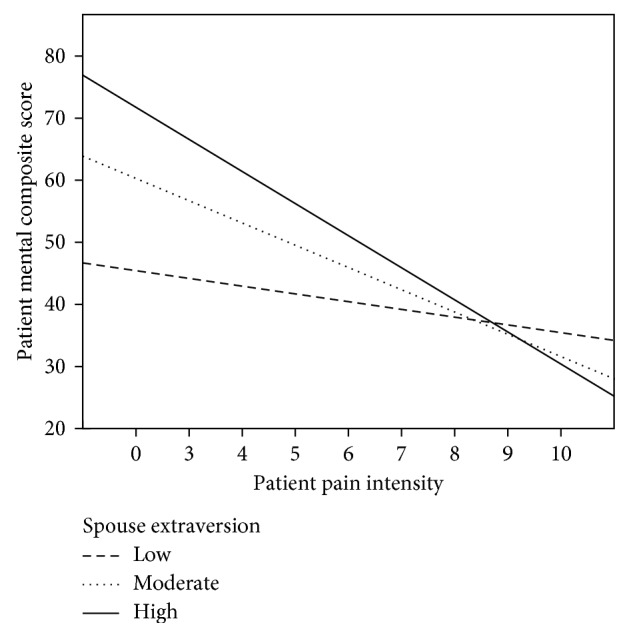
Extraversion as a moderator of the pain intensity to mental health relationship.

The post hoc probing with extraversion as a moderator (Figure 2) showed similar results to those obtained for neuroticism. Specifically, pain intensity was not significantly associated with mental health for participants with an introverted (low in extraversion) spouse (*β* = −0.15, *t* = −1.5, *p*=0.115; 95% CI = −2.38, 0.26). On the contrary, pain intensity was significantly related to mental health when the spouse extraversion was either high (*β* = −0.34, *t* = −2.43, *p*=0.019; 95% CI = −6.58, −0.61) or moderate (*β* = −0.53, *t* = −3.22, *p*=0.003; 95% CI = −8.46, −1.87).

Multiple regression diagnostics revealed no problems of model fit to the data. Specifically, the variance inflation factor, which reveals to what extent the variance is inflated due to multicollinearity, was lower than 2 for all predictors. To test the existence of influential cases, we assessed how the regression coefficients changed by excluding an observation by means of the standardized DFBETA, with a particular interest in the interaction coefficient. All values were smaller than 1, suggesting that none of the observations substantially influenced the model [41].

## 4. Discussion

The present investigation aimed at exploring the moderating role of spouse personality in the relationship between pain intensity and health-related quality of life in patients with chronic pain. Based on previous research showing pain intensity and certain personality dispositions (i.e., neuroticism, introversion, and low conscientiousness) associated with poor health outcomes [4, 9], we expected that spouse neuroticism, introversion, and low conscientiousness would add to the burden of pain by imposing poor health across pain levels. Our results partially support our hypothesis. On the one hand, the predicted moderation occurred for spouse neuroticism and extraversion. On the other hand, these findings were not replicated for conscientiousness, and the moderation of neuroticism and extraversion only occurred for physical and mental components of health-related quality of life, respectively.

Research has repeatedly shown that experiencing pain impacts negatively on the physical and mental health status of individuals [42–45]. In our study, this was replicated when spouse neuroticism and introversion were low or moderate (i.e., health-related quality of life decreased with pain). However, pain intensity was weakly or nonsignificantly associated with health outcomes when spouse neuroticism or spouse introversion were high, indicating similar levels of (low) functioning irrespective of the intensity of the stressor (pain) and suggesting that spouse personality adds to the burden of pain on health-related quality of life, especially at lower levels of pain intensity. A mechanism by which spouse personality might influence pain-health associations is proposed in accordance with the Transactional Model Of Health [18], although this remains speculative and further conclusions cannot be drawn from the present study results.

The Transactional Model Of Health and more recent discussions on interpersonal models of health in chronic pain [7, 18] propose that spouse appraisal might enhance similar cognitive patterns in patients, ultimately explaining patient behavior and health-related quality of life. Individuals scoring high in neuroticism tend to be fearful and preoccupied and report poor mental and physical health status [46–48]. Similarly, spouses' worry and negativity towards the patient's pain behavior has been associated with impaired physical and mental health of pain patients [17, 49]. According to the Transactional Model of Health, the moderating effect of spouse neuroticism revealed in the present investigation would be explained by means of modeling mechanism by which spouse preoccupation and worry would enhance similar affective and cognitive reactions in patient. These affective states are known to lead to maladaptive behaviors (i.e., avoidance of movement and disuse of painful body parts due to fear) and, ultimately, to impaired physical status [50], which would be consistent with our findings.

In our study, pain-to-health associations in the patient also varied as a function of spouse extraversion. Specifically, patients whose spouses presented low levels of extraversion (i.e., introversion) showed poor mental health irrespective of the intensity of their pain. Research in chronic pain has indicated that optimism, social interactions, and physical activities are key factors associated with the mental well-being of pain patients [23, 51–53]. However, evidence suggests that individuals low in extraversion are not very physically active, pessimistic, and have little interest for social interactions [9, 54]. Again, though this is only a hypothesis, a possible mechanism explaining these results is provided in accordance with the Transactional Model of Health. Specifically, it is possible that introverted spouses may add to the patients' mental burden when experiencing lower levels of pain by stimulating pessimistic thoughts about the disease and failing to promote the use of beneficial coping efforts, such as social interactions and physical activity.

Regarding the other personality dimensions, our study revealed that spouse openness to experience, agreeableness, and conscientiousness did not moderate the pain-health relationship in the pain patient. Research has previously shown that the contribution of openness and agreeableness to well-being is small when compared with that of neuroticism, extraversion, and conscientiousness [9, 55, 56]. The fact that conscientiousness did not emerge as a significant moderator of the pain-health relation is surprising in light of the role of this personality dimension in health settings [11]. However, its role in chronic pain settings has been argued to be modest [57–59], so its contribution might not be generalizable to all populations.

In our study, spouse neuroticism moderated pain-to-physical health associations, while the moderation of extraversion occurred for mental health. Extraversion is frequently associated with better physical and mental health in the general population [9]. However, its relation with physical health in pain settings appears to be less clear [57, 60], which might explain the results in our study. It is less clear why neuroticism of the spouse only correlated to physical and not to mental health. Most studies show a negative association between neuroticism and mental health [8, 57], so one would also expect a moderating effect neuroticism on pain-to-mental health associations. In fact, there is previous evidence to suggest that the relationship between a stressor (i.e., everyday problems) and both affective and physical problems are moderated by spouse neuroticism [19]. While the present study replicated this moderating effect of spouse neuroticism on physical health status, this was not the case for mental well-being.

The study of spouse personality, the inclusion of important covariates of patient health (i.e., patient personality and demographic factors), and the analysis of moderation are some of the strengths of the present investigation. Previous research had explored the association between spouse behaviors (i.e., response styles) and several outcomes in the patient, including pain intensity reports and physical and mental health status [16, 61, 62]. However, the role of spouse personality, as well as the moderating effect of spouse characteristics remained unexplored. The present study adds to the existent literature by showing that a possible mechanism by which spouse factors (i.e., personality) might contribute to patient health is by exacerbating or attenuating its relationship with pain intensity. Also importantly, moderation occurred even after controlling for patient demographic and personality characteristics.

The present study is not without limitations. Although some mechanisms explaining the moderation effects were proposed, they should be considered as hypotheses and need further investigation. Also, the effect sizes of the moderator were low. Although this is frequent in moderation studies, the power of the associations should not be overestimated. Additionally, some factors that might be important for the mental well-being of couples, such as length and quality of marriage, were not assessed in this investigation. In fact, while being married was a key eligibility factor in the study, the quality of the relationship, even without marriage, might be an interesting factor to be considered in the present study, as well as in future, similar investigations. Finally, there are methodological limitations in this study too. For example, the design used prevents us from drawing any causal relationship from our findings and do not allow us to determine the direction of the associations. Also, the exclusion of unmarried couples inevitably affects the generalizability of findings.

## 5. Conclusions

While acknowledging the aforementioned shortcomings, our study might be important for applied settings: first, because patient and spouse characteristics in this study (patient health status and pain intensity and patient and spouse personality) were comparable to those of previous investigations, thus supporting the generalizability of the results; also, because the results revealed that the relationship between a chronic stressor (i.e., chronic pain) and health status may be complex. Specifically, our results suggest that this relationship may be influenced by contextual variables (i.e., spouse personality), as suggested in previous research [63, 64]. Therefore, it would be interesting to see whether a reduction of situational demands (i.e., pain intensity) before couple-oriented interventions maximizes the positive effects of treatments.

## Figures and Tables

**Table 1:** Means and standard deviations of patient and spouse characteristics.

	Patient	Spouse
Age	56.82 (13.60)	56.66 (13.85)
Pain duration (years)	6.56 (8.57)	
*Personality*		
Neuroticism	20.05 (8.45)	24.19 (7.05)
Extraversion	26.99 (7.54)	31.79 (5.53)
Conscientiousness	32.43 (6.87)	24.54 (8.96)
Openness	31.86 (6.84)	26.26 (8.08)
Agreeableness	30.88 (6.20)	22.74 (7.41)
*Pain and health status*		
Pain intensity	7.71 (1.56)	
Physical Functioning	34.80 (23.65)	
Mental Composite Score	39.04 (12.44)	

**Table 2:** Moderation analysis of neuroticism in the relationship between pain intensity and Physical Functioning.

Block	Independent variables	*β*	95% CI	*t*	*p*	*R* ^2^	*F*	*p*
1	Patient age	−0.22	−0.62, −0.13	−3.01	0.003	0.104	4.05	<0.001
	Patient sex	<0.01	−6.24, 6.40	0.02	0.981			
	Pain duration	−0.08	−0.70, 0.17	−1.21	0.229			
	Educational level	0.03	−4.93, 8.14	0.49	0.628			
	Patient N	−0.01	−0.43, 0.37	−0.13	0.899			
	Patient E	0.15	0.03, 0.87	2.12	0.035			
	Patient C	0.06	−0.28, 0.66	0.80	0.424			
2	Patient pain intensity	−0.45	−8.94, −4.73	−6.41	<0.001	0.138	30.25	<0.001
3	Spouse N	−0.01	−0.35, 0.39	0.12	0.907	<0.001	<0.01	0.969
4	Spouse N ∗ patient pain	0.19	0.08, 0.54	2.65	0.009	0.021	7.01	0.009

*Note. R* ^2^ is adjusted. *R*^2^ and *F* refer to changes in each block. Reported beta values are standardized and correspond to the final model. N, neuroticism; E, extraversion; C, conscientiousness.

**Table 3:** Moderation analysis of extraversion in the pain-mental health relationship.

Block	Independent variables	*β*	95% CI	*t*	*p*	*R* ^2^	*F*	*p*
1	Patient age	0.08	−0.05, 0.20	1.17	0.244	0.265	10.50	<0.001
	Patient sex	−0.06	−4.67, 1.60	−0.97	0.334			
	Pain duration	−0.06	−0.33, 0.11	−0.95	0.341			
	Educational level	0.10	−0.89, 5.79	1.45	0.150			
	Patient N	−0.36	−0.70, −0.30	−4.86	<0.001			
	Patient E	0.11	−0.04, 0.38	1.58	0.116			
	Patient C	0.10	−0.07, 0.41	1.39	0.166			
2	Patient pain intensity	−0.25	−3.03, −0.90	−3.65	<0.001	0.030	8.37	0.004
3	Spouse E	0.02	−0.18, 0.24	0.29	0.770	<0.001	<0.01	0.995
4	Spouse E ∗ patient pain	−0.17	−0.30, −0.03	−2.47	0.014	0.016	6.10	0.014

*Note. R* ^2^ is adjusted. *R*^2^ and *F* refer to changes in each block. Reported beta values are standardized and correspond to the final model. N, neuroticism; E, extraversion; C, conscientiousness.

## Data Availability

The datasets generated and analyzed during the current study are not publicly available due individual privacy but are available from the corresponding author on reasonable request.

## Abbreviations

FFM: Five-factor model
NEO-FFI: NEO five-factor inventory.
